# Assessing the structural conservation of protein pockets to study functional and allosteric sites: implications for drug discovery

**DOI:** 10.1186/1472-6807-10-9

**Published:** 2010-03-31

**Authors:** Alejandro Panjkovich, Xavier Daura

**Affiliations:** 1 Institute of Biotechnology and Biomedicine (IBB), Universitat Autònoma de Barcelona (UAB), Bellaterra, E-08193, Spain; 2 Catalan Institution for Research and Advanced Studies (ICREA), Barcelona, E-08010, Spain

## Abstract

**Background:**

With the classical, active-site oriented drug-development approach reaching its limits, protein ligand-binding sites in general and allosteric sites in particular are increasingly attracting the interest of medicinal chemists in the search for new types of targets and strategies to drug development. Given that allostery represents one of the most common and powerful means to regulate protein function, the traditional drug discovery approach of targeting active sites can be extended by targeting allosteric or regulatory protein pockets that may allow the discovery of not only novel drug-like inhibitors, but activators as well. The wealth of available protein structural data can be exploited to further increase our understanding of allosterism, which in turn may have therapeutic applications. A first step in this direction is to identify and characterize putative effector sites that may be present in already available structural data.

**Results:**

We performed a large-scale study of protein cavities as potential allosteric and functional sites, by integrating publicly available information on protein sequences, structures and active sites for more than a thousand protein families. By identifying common pockets across different structures of the same protein family we developed a method to measure the pocket's structural conservation. The method was first parameterized using known active sites. We characterized the predicted pockets in terms of sequence and structural conservation, backbone flexibility and electrostatic potential. Although these different measures do not tend to correlate, their combination is useful in selecting functional and regulatory sites, as a detailed analysis of a handful of protein families shows. We finally estimated the numbers of potential allosteric or regulatory pockets that may be present in the data set, finding that pockets with putative functional and effector characteristics are widespread across protein families.

**Conclusions:**

Our results show that structurally conserved pockets are a common feature of protein families. The structural conservation of protein pockets, combined with other characteristics, can be exploited in drug discovery procedures, in particular for the selection of the most appropriate target protein and pocket for the design of drugs against entire protein families or subfamilies (*e.g. *for the development of broad-spectrum antimicrobials) or against a specific protein (*e.g. *in attempting to reduce side effects).

## Background

Molecular processes in the living cell are coordinated and executed under tight regulation. Proteins play a fundamental role in almost all biological processes, and their overall activity is regulated at different levels [1]. At a first level, the concentration of a particular protein in the cell is regulated through its synthesis rate (gene expression) and its degradation rate. At another level, mechanisms act on the protein molecule itself through covalent modifications or non-covalent binding of small ligands or other molecules. These regulatory mechanisms are not only essential for the proper functioning of the molecular processes that maintain life, but are also responsible for cross-signaling and regulation processes between an organism and its environment.

Many metabolic enzymes, signalling proteins and transcription factors, among others, are regulated allosterically. Allosteric regulation has been studied for more than 50 years and it is considered the most powerful and common way to regulate protein activity [2]. However, for most known cases of allosterism, the atomic details that explain the functional relationship between distant sites on the same protein molecule have not been elucidated [3,4].

Many pharmaceutical compounds act through allosteric regulation, as seen in the case of paclitaxel (Paxol), a cancer therapeutic drug that regulates tubulin polymerization allosterically [5,6]. Even though active sites represent the classic drug-target pocket (*e.g. *Aspirin and cyclooxygenase), allosteric sites present advantages over active sites in the context of drug design. Enzymatic activity usually involves charged transition states and the substrates are not always drug-like. Thus, orally active inhibitors that complement these sites can be very difficult to obtain. Moreover, allosteric sites may allow the discovery of not only novel drug-like inhibitors, but activators as well [2,3].

In this context, predicting allosteric sites computationally is of great interest. Allosteric sites have been predicted using structural information [7] and phylogeny [8]. Recently, methods have been developed in order to model or predict the relationship between allosteric and active sites [9-11]. These methods represent an important step forward in the understanding of allosterism. However, these studies are limited by the low quantity of readily available data on allosteric sites. As stated by Thornton and collaborators in their recent review [4], this is due in part to the lack of a formal database that organizes and stores knowledge on allosteric proteins and the corresponding mechanisms.

To unveil common patterns underlying allosterism, given that these exist, a large-scale study using structural and sequence data would be necessary. However, given the present scenario of scarce allosteric-site data, we decided to perform a large-scale analysis of protein ligand-binding pockets, as these represent potential locations of functional and allosteric or regulatory sites. Our approach is supported by the concept that besides naturally ocurring allosteric sites, serendipitous sites -having no natural ligand but effectively being an allosteric site given an appropriate ligand- may be of great pharmacological interest [2]. Examples of previously unknown allosteric sites discovered on already solved protein structures [12,13] support the idea that orphan or serendipitous allosteric sites exist which may lack a known natural effector, but provide an excellent opportunity for drug discovery approches such as virtual screening. Hardy and Wells also suggest that the large amount of 'crystallization artifacts' present in the Protein Data Bank (PDB) [14], such as ligands co-crystallized in unexpected binding sites, could hint the presence of previously unknown allosteric sites [2].

A large database of protein structures and associated small-molecule ligands is available [15] and has been used to predict ligand-binding sites by homology [16]. However, small-molecule ligands are not always easy to co-crystallize and we did not want to limit our study to only such cases. In this context, ligand-binding sites can be computationally predicted from structure alone with reasonable accuracy [17-20]. To our knowledge, ligand-binding pockets as predicted directly from structure [19] have not been studied or characterized at large-scale yet, even though they represent the potential location of yet unknown effectors [2].

Functional pockets in proteins have been previously characterized in terms of their flexibility [21,22], evolutionary conservation [21,23] and electrostatic potential [24] and these characteristics have been used to predict their presence and location in the protein structure [23]. Evolutionary conservation is a common characteristic of biologically functional sites. However, until now it has been exploited solely at the sequence level [23]. Although sequence and structural conservation correlate, structure is closer to function and may be conserved even in the lack of a sequence-level signal [25]. Despite this, to our knowledge, an approach based on the structural conservation of protein pockets has not been previously used. Here, we introduce a simple methodology to study pockets at the protein family level, consisting in the identification of pockets present in equivalent positions across different structures of the same protein family. To parameterize the method, we used protein pockets that matched known active sites, as these are well annotated [26,27]. Once parameterized, we applied the method to all protein structures available in the PDB [14], leading to the identification of protein pockets for thousands of different protein families [26]. Next, we compared the levels of structural conservation with other pocket characteristics estimated on the same protein families, such as sequence level conservation, backbone flexibility and electrostatic potential.

In the following sections we also discuss the results of this analysis for a small set of biological examples which illustrate the relevance of structural conservation in studying protein functional and regulatory sites. Finally, we perform an estimation of the amount of potentially paired regulatory and functional sites that may exist in the entire data set.

## Results and Discussion

### Initial structural data set

To acquire a large-scale perspective on the conservation of protein pockets, we gathered all available protein structures from the Protein Data Bank (PDB) [14]. We applied a set of filtering criteria to ensure the quality and relevance of the structural data before grouping the structures by protein families, as defined by the Protein families database (Pfam) [26]. To partially cope with the inherent bias present in the PDB, where proteins tend to be over- or under-represented [28], we selected a set of representative structures for each protein family (see Methods). The final data set covered 4,258 different Pfam protein families and was composed of a total of 22,312 distinct protein structures (maximum 95% sequence identity), on which we predicted the location of 167,648 putative ligand-binding pockets by means of the LIGSITEcs program [19].

### Identifying equivalent pockets across different protein structures

The first step to estimate the structural conservation of protein pockets was to identify those that appeared at equivalent positions in different structures of the same family. Briefly, for each protein family the pockets predicted for a representative set of structurally aligned proteins [29] were clustered following the approach described in the Methods section. The clustering method requires a threshold distance to select equivalent pockets across superimposed structures. After visual inspection of preliminary results, we observed that this parameter would be related to the structural fluctuation present in each protein family. We decided to use known active sites as a reference to define this parameter, as we were able to map a total of 8,046 pockets (covering 319 distinct protein families) to Pfam-annotated or predicted active-site residues unambiguously (see Methods). If the active site is well conserved across the whole protein family, an ideal clustering method would include all active sites of the different structures in the same cluster (true positives), without including any non-active site pockets (false positives). After benchmarking a range of different values (see Table 1), we defined the family-specific distance threshold to be 2.0 Å plus the average RMSD observed when superimposing the representative structures of the protein family. This approach showed a good compromise between true positives (including an average of 76.5% of all active sites) and false positives (including 8.95% of non-active site pockets), as shown in Table 1. For the families included in this study, the average value observed for the family-specific threshold was 4.5 Å.

**Table 1:** Parameterization. Performance of the clustering algorithm when grouping known active sites.

Fixed distance value (Å)	% total active sites	% non active sites
0.0	59.60	4.46
1.0	69.89	5.31
2.0	76.47	8.95
3.0	79.13	13.67
4.0	82.78	17.30
5.0	83.50	23.04
6.0	86.71	27.90
7.0	87.43	34.68
8.0	87.62	41.97
9.0	88.24	49.59
10.0	89.29	56.87

### Assessing the structural conservation of pockets on protein families

**Figure 1:**
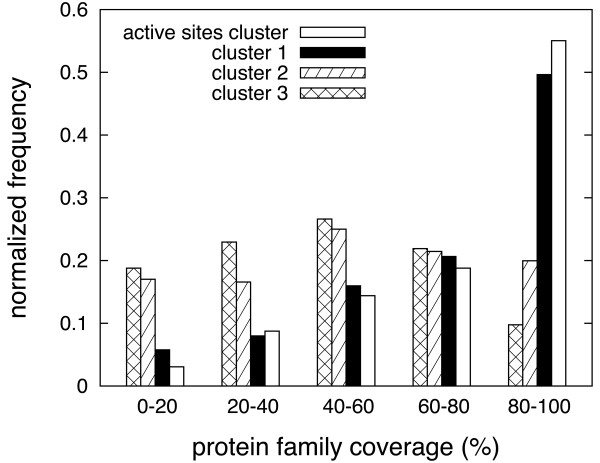
**Structural conservation**. Histograms displaying protein family coverage of different pocket clusters, for a total of 1,128 protein families with five or more representatives in the data set.

After parameterizing the clustering method using active sites as reference, we applied it to all protein families having at least 5 representative structures in the data set (a total of 1,128 protein families satisfied this requisite). We then analyzed the resulting clusters of pockets in terms of the percentage of representative structures covered by each cluster. A very well conserved pocket would be expected to appear in all representative structures of the protein family, *i.e. *100% coverage. Thus, this coverage can be taken (and will be taken throughout this study) as a measure of the pocket's structural conservation within the protein family. This analysis was performed, for each family, for the first three clusters and for the cluster containing the largest amount of active sites (active-site cluster). The results are illustrated in Figure 1. Note that cluster ranking is based on average pocket size and coverage as described in the Methods, and that the active site cluster overlaps with the 1st, 2nd and 3rd clusters in 117, 38 and 13 families, respectively.

According to the histograms in this figure, the higher the coverage of the active site cluster the higher its frequency in the ensemble of families. This, which should be expected for pockets that are functionally relevant at the family level, is also applicable to the distribution of cluster 1 but not to those of clusters 2 and 3. Yet, the coverage distributions of clusters 2 and 3 suggest that they could be important at a sub-family level, remaining compatible with an allosteric function which may have different faces within the same protein family. In global terms, the average coverage of the first cluster or most conserved pocket of the 1,128 protein families analyzed is 85%. Of these 1,128 protein families, 398 (35%) show at least one pocket cluster that covers 100% of the protein-family representatives, while 884 (78%) present a pocket cluster that covers at least 75% of the protein family.

These results show that for the majority of the protein families analyzed there is at least one pocket with high levels of structural conservation. We expected a high frequency of conserved pockets among enzymes, but not all protein families in the data set have been annotated with a biological activity that is related to a pocket in the protein structure. A structurally conserved pocket whose biological function has not been described is an optimal candidate for further computational and experimental analysis. For example, in the context of drug design and discovery, the information on whether a pocket on the target protein is structurally conserved or not may be useful when designing a wide-spectrum or a specific drug, respectively, and in choosing the appropriate ligand-binding site for virtual screening. Clearly a pocket that is very well conserved at the structural level may not necessarily have functional properties but be the consequence of structural restraints common across the protein family. Nevertheless, it may still be of interest to explore its possible exploitation as a serendipitous allosteric site for a therapeutic application [2].

Before further exploring these possibilies, we analyzed the degree of correlation between structural conservation and other properties often used for the characterization of protein pockets, such as evolutionary conservation at the sequence level [18,21,23], protein flexibility [21,22] and electrostatic potential [24]. These parameters may be useful in distinguishing pockets that are conserved because of their biological function from pockets that are conserved because of structural restraints.

### Comparison with other pocket characteristics

#### Sequence conservation

Biologically relevant residues tend to be conserved at the sequence level [30]. In this context, the degree of conservation of the residues defining a protein cavity may be taken as a measure of the cavity's conservation [18-20]. The statistical significance of this measure can be then tested by comparing the levels of sequence conservation in the pocket and in the rest of the protein (see Methods).

**Figure 2:**
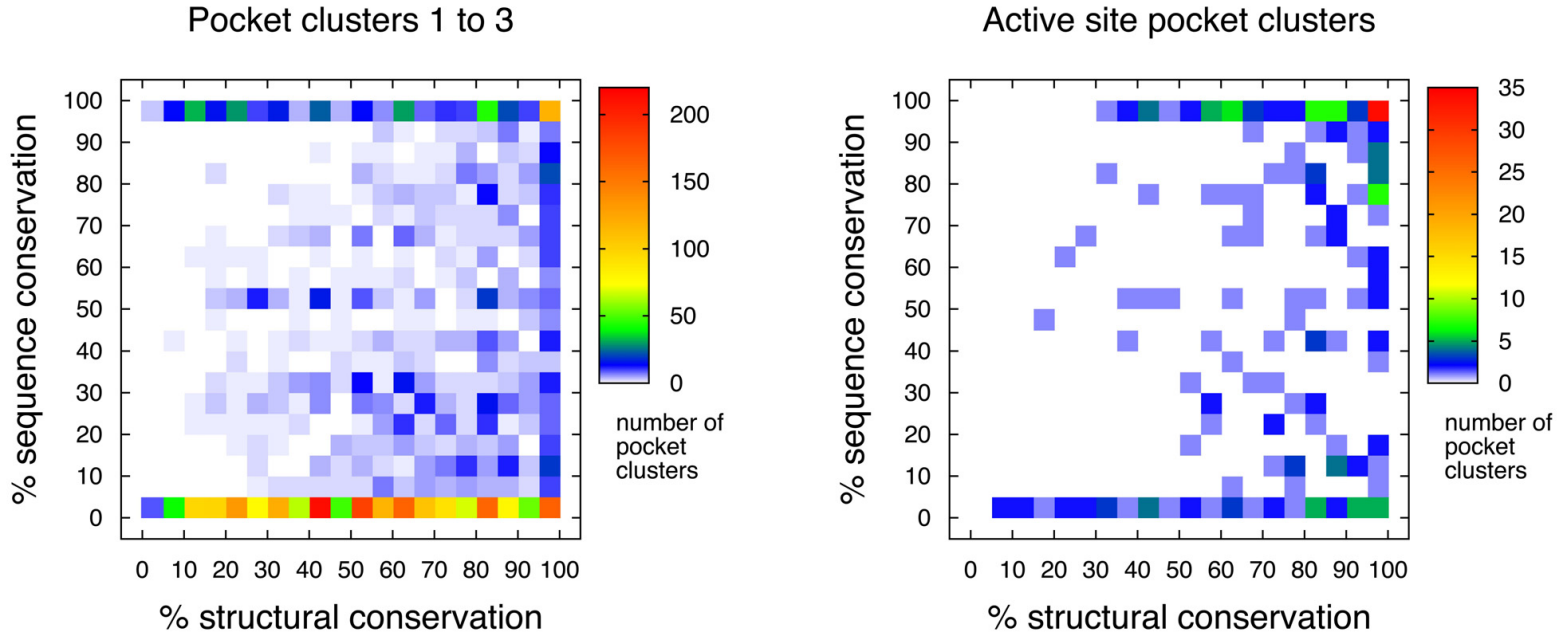
**Sequence conservation**. Two-dimensional histograms comparing structural and sequence conservation for pocket clusters of the different families in our data set. The left panel displays the values for pocket clusters 1 to 3 of 1,128 distinct protein families and the right panel shows the distribution for clusters containing the majority of active sites for 229 protein families. Only families with at least five representative structures are included in the analysis.

Although structure is in general more conserved than sequence [25], the two characteristics are related. To analyze the relationship between sequence and structural conservation for pocket clusters across protein families, we quantified sequence conservation as the percentage of pockets in the cluster that are significantly conserved at the sequence level. The structural and sequence conservation values for clusters 1 to 3 of all protein families with at least five representative structures are compared in the left panel of Figure 2. It is shown that there is a relatively small correlation between sequence conservation and structural conservation of the pockets, with the highest density (62% of the population) at the 0-5% sequence-conservation end of the distribution. Yet sequence conservation is clearly peaked also at the 95-100% end (12% of the population), indicating that in general structurally conserved pockets (pocket clusters) may be well conserved at the sequence level, or not at all, leaving few cases in between.

One may argue that pocket clusters displaying high sequence and structural conservation may match biologically functional pockets across the protein family, while clusters displaying only structural conservation may often play a purely structural role. In relation to this, and giving the conservation percentages the right context, it should be noted that a large proportion of the protein families included in this analysis may not present a biological activity that is related to a particular pocket in the structure. Nevertheless, some pockets may be biologically relevant, despite a lack of sequence conservation. An example of this is given by L-lactate dehy-drogenase (LDH), for which the allosteric site [31] is very well conserved at the structural level (89.8%), but shows no signal of sequence conservation (0.0%) in this analysis. We describe the LDH case in further detail below.

As discussed above, pockets that are conserved at the structural level but have not been previously described as ligand-binding sites may be evaluated as potential orphan or serendipitous allosteric sites and targeted for drug discovery and design. The low sequence conservation we observed in many of the structurally conserved pockets indicates that even though the pocket is detected in the same location, the residues defining the pocket are not under direct evolutionary pressure and may vary in type. This variation in residue composition could aid the design of highly specific drugs that would bind only certain members of the protein family.

We performed the same distribution analysis for pocket clusters that included the largest amount of active sites in the corresponding protein families, with results displayed in the right panel of Figure 2. In this case, the largest population corresponds to high levels of both sequence and structural conservation, as expected, although numerous exceptions appear in this set as well. Some exceptions rise because not all members of a given Pfam protein family may be enzymatically active (*e.g. *Globin).

Note that the level of sequence conservation of each cluster is estimated from its member pockets and is independent of the weight of the cluster in the set of family-representative structures. A sequence conservation of 100% means that every single pocket in the cluster is significantly conserved at the sequence level, although the cluster may only cover half of the protein family, *i.e. *50% structural conservation. This is also valid for the flexibility and electrostatic-potential analyses described below.

#### Flexibility

Protein function is fundamentally linked to dynamics. In this context, the properties of a protein's ligand-binding site are to an important extent a function of the site's flexibility, entropy being an essential component of the free energy of binding. Thus, relatively small changes in flexibility often have a large effect on ligand-binding affinites [32]. Moreover, some allosteric sites regulate protein function by modifying the protein's flexibility upon ligand binding [2,9,33].

Flexibility may be estimated on a residue basis from structural B factors. This has been previously used, for example, to show that active sites tend to be more rigid than the rest of the enzyme structure [21,22].

**Figure 3:**
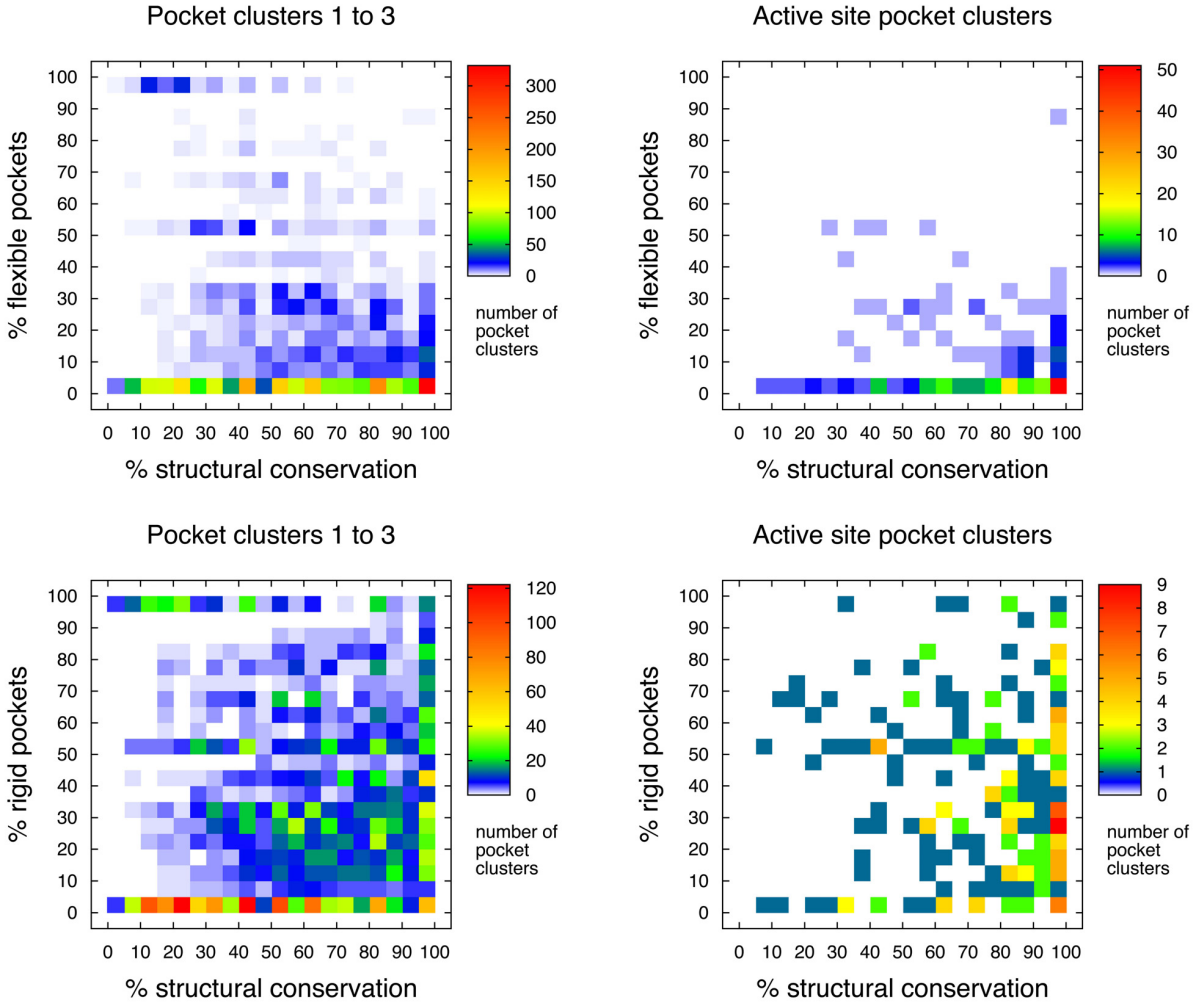
**Flexibility**. Two-dimensional histograms comparing structural conservation and flexibility for pocket clusters of the different families in the data set. The panels on the left display the values for pocket clusters 1 to 3 of 1,128 distinct protein families and the right panels show the distribution for clusters containing the majority of active sites for 229 protein families. The top panels show the percentage of pockets that were significantly flexible when compared to the rest of the structure, while the bottom panels show the percentage of pockets found to be significantly rigid (less flexible) than the rest of the corresponding protein structure. These plots only include protein families with at least five representative structures.

We analyzed the flexibility of residues forming part of pockets and determined if they showed significantly higher or lower values of flexibility than the rest of the protein's backbone, classifying them as 'flexible' or 'rigid', respectively, as described in the Methods section. We then compared the percentage of significantly rigid or flexible pockets found in the different pocket clusters with their structural conservation. The results are illustrated in Figure 3. These results show very few cases where pockets are significantly more flexible than the rest of the protein, and these few cases (clusters 1-3, left panel) tend to be poorly conserved in terms of structure. However, the structural conservation of a very flexible pocket would be probably hard to quantify by our method, given that a large degree of structural variation would to some extent impede its detection across different proteins of the same family. Even in average cases, member pockets of the same cluster can display large differences in shape and volume, as seen in the case of LDH described below.

The lower panels of Figure 3 show a wide distribution of significantly rigid pockets. This means that within a family, the levels of flexibility for the same pocket may differ from structure to structure considerably. These results were expected to a certain level, since flexibility may vary under different experimental conditions of structure determination and it may as well be modified by the presence of bound ligands or other proteins [32]. In the case of active site pocket clusters, the lower-right panel of Figure 3 displays a stronger signal for structural conservation than for rigidity.

Protein flexibility is a major issue for ligand virtual screening and design [34]. Although key residues in active sites, such as those involved in catalysis, tend to be rigid [21,22], they coexist with regions of high flexibility, which are necessary to allow for ligand exchange. When searching for other possible ligand-binding sites for the screening and design of effector molecules, one will usually target pockets that are sufficently flexible that binding will not be blocked by high free-energy barriers (involving conformational rearrangements) but at the same time sufficiently rigid that computational docking will be reliable and that there will not be a sizable entropic penalty due to a potentialy large loss of flexibility upon ligand binding (which would need to be compensated enthalpically for effective binding). The analysis shown here might provide a basis to select structurally conserved pockets with specific flexibility properties.

#### Electrostatic potential

**Figure 4:**
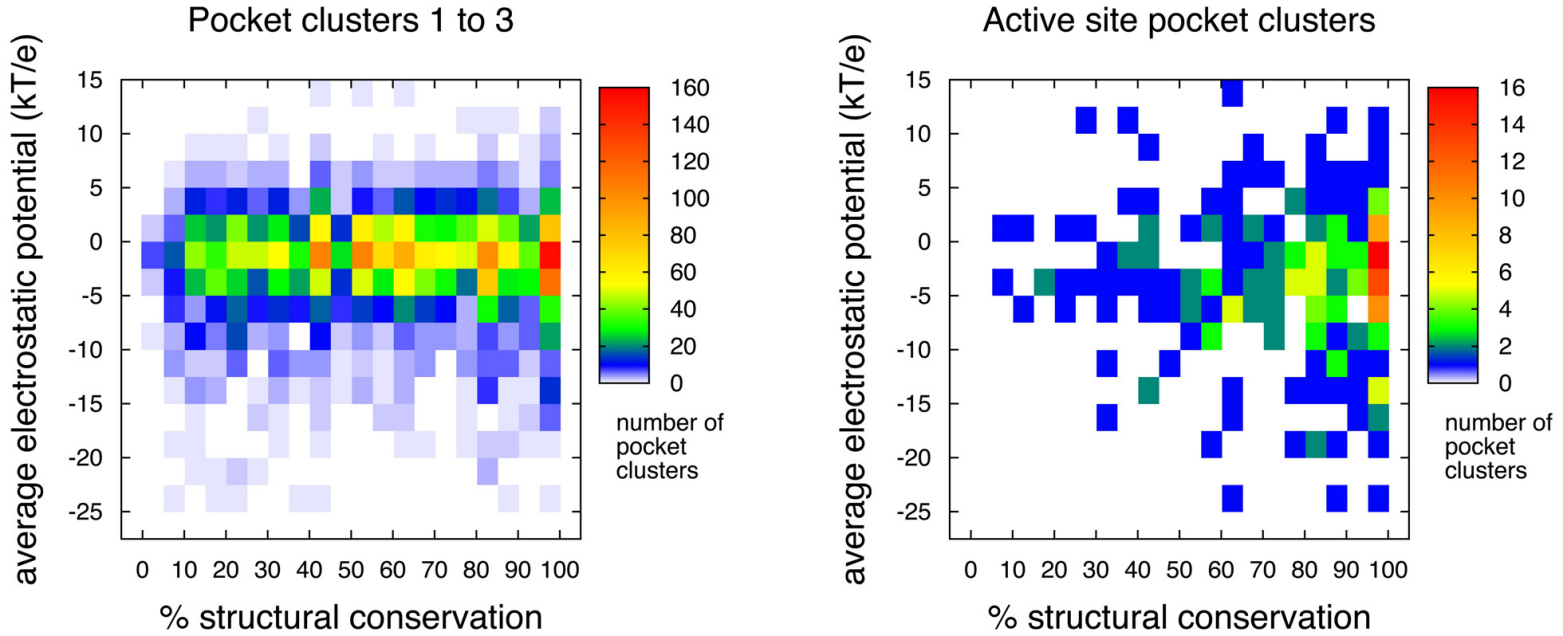
**Electrostatic potential**. Two-dimensional histograms comparing structural conservation and electrostatic potential for pocket clusters of the different families in the data set. The panels on the left display the values for pocket clusters 1 to 3 of 1,128 distinct protein families and the right panels show the distribution for clusters containing the majority of active sites for 229 protein families. These plots only include protein families with at least five representative structures.

The electrostatic potential, as estimated by solving the Poisson-Boltzmann equation for protein structures with force-field-based charge distributions [35] has been previously used to characterize and predict enzymatic active sites [24]. For each pocket in the data set we estimated the electrostatic potential at the pocket's center of mass as described in the Methods section and computed the average value over the pockets for each of the first three pocket clusters in every protein family. The combined distribution of average electrostatic potential and structural conservation of pocket clusters is shown in Figure 4. Clearly, this property does not correlate either with structural conservation. Most values cluster between -5 and 2.5 *kT/e*, even for active site pockets. However, this measure is probably the least conserved across the different pockets of a given cluster. In fact, for 42.4% of the pocket clusters included in the left panel of Figure 4, the standard deviation is larger than the average absolute value. It appears that the pocket's electrostatic potential, as estimated here, is largely protein specific, and that this measure is hard to extrapolate across the different proteins in a family. Nevertheless, the values of electrostatic potential can still be used in refining the selection of pockets for drug-screening procedures, given that drug-like ligands may be easier to find for more neutral sites than for strongly charged or polarized pockets [2]. These values could also be used to distinguish putative active sites from allosteric sites in the lack of proper annotation (see PIG-L below).

### Biological examples

To complement the large-scale perspective presented above we analyzed a few protein families in more detail. The examples described below emphasize the relevance of structural conservation in the study of allosteric and functional protein pockets.

#### L-lactate dehydrogenase

**Figure 5:**
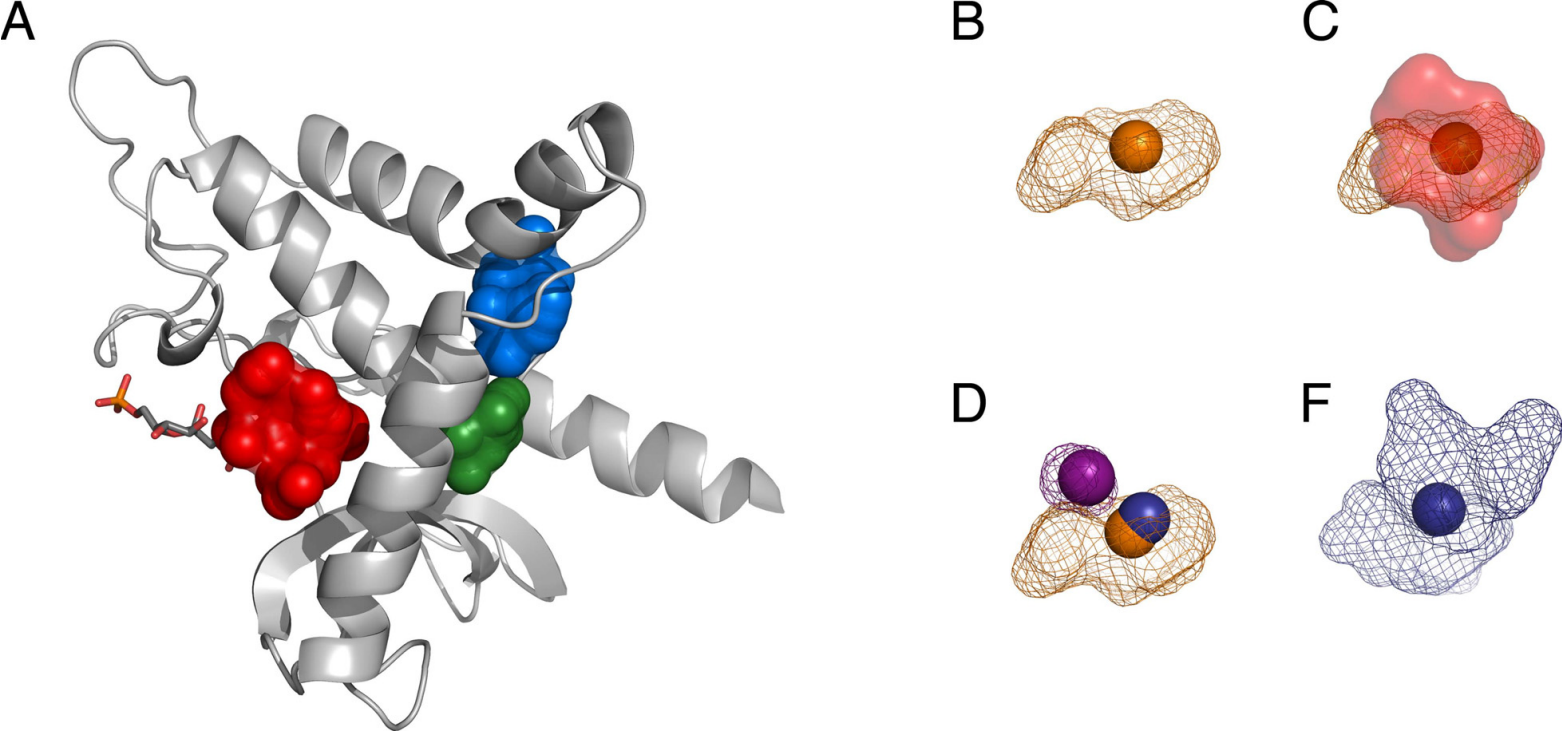
**LDH pocket clusters**. A) LDH structure ([PDB:1LTH], chain R, residues 150-317 which correspond to Pfam Ldh_1_C domain). Pocket clusters are displayed as the centers of mass of member pockets in 'spheres' representation (cluster 1: red, 2: green, 4: blue). The allosteric effector fructose 1,6-bisphosphate is displayed in 'sticks' representation next to cluster 1. Clusters 2 and 4 match the active site of LDH, while cluster 1 matches the allosteric site. B) Member pocket of cluster 1, found in structure [PDB:1SOV]. The center of mass of the pocket is displayed in 'spheres' representation while the volume of the actual cavity is shown in 'mesh' representation, both in orange color. C) The member pocket displayed in 'B' is shown in its relative position to the allosteric site cluster 1. Cluster 1 is shown in semi-transparent red 'surface' representation, similar as in A). D) The cluster 1 member pocket of [PDB:1SOV] displayed in orange in its relative position to other member pockets of the same cluster 1. The member pocket in purple was predicted on structure [PDB:1MLD], while the blue 'sphere' represents the center of mass of the member pocket found in yet another structure ([PDB:2A92]). F) Detail on the shape of the member pocket found in structure [PDB:2A92]. Structural orientations were kept constant (after protein backbone superimposition) to illustrate the varied shapes and volumes that member pockets of a single pocket cluster may display.

L-lactate dehydrogenase (LDH) catalyzes the reduction of pyruvate by NADH to L-lactate in the last step of glycolysis. Certain bacterial LDHs, in contrast to their mammalian counterparts, display allosteric regulation by fructose 1,6-bisphosphate (FBP) [36]. Iwata and co-workers solved the structure of LDH ([PDB:1LTH]) in both active (R) and inactive (T) states, co-crystallized with the allosteric activator [31]. The Ldh_1_C domain in the R state (relaxed or active) of LDH is displayed in Figure 5A with the bound allosteric activator and pocket clusters 1, 2 and 4, as calculated for this family. Figures 5B-F show examples of distinct member pockets matching cluster 1 in the LDH protein family.

The active site in this protein matches pocket clusters 2 and 4. Both clusters are very well conserved at the sequence level with, respectively, 92.1% and 77.8% of the included pockets being significantly conserved. These active-site pockets are also well conserved at the structural level: cluster 2 appears on 75.5% of the representative structures while cluster 4 appears on 65.3% and, when considered together, at least one of them appears on 94% of the protein family. Interestingly, the pocket cluster with the highest structural conservation (cluster 1), corresponds to the allosteric site (Figure 5). In this case the allosteric cluster covers the majority of representative structures for this family (89.8%). However, the average sequence conservation signal is very low (-0.12) and we found none of the 51 pockets included in this cluster to be significantly conserved at the sequence level. This means that an evolutionary analysis based purely on sequence information would not find this site to be significally conserved, while the structure-based approach points it out as the most conserved pocket in this protein family.

The allosteric site cluster is remarkable in terms of flexibility as well, with 81.2% of included pockets being significantly rigid (see Methods). In the case of PDB entry [PDB:1LTH], there is a clear difference in the global flexibility values we calculated for the R (0.43) and T (-0.56) structures of the protein, corresponding to the active and inactive states, respectively. However, the allosteric site pocket shows consistently low values, -0.52 and -0.93 for R and T, respectively, with both pockets being significantly rigid according to the statistical test (*p*-values of 0.0008 and 0.0012, respectively). The active site in [PDB:1LTH] shows no significant differences in terms of flexibility when compared to the rest of the structure, although as expected, differences between the T and R states are also observed. The rigidity of the active site pockets through the whole family is not clear from the data, as 69.4% and 25.7% of the pockets are significantly rigid for clusters 2 and 4, respectively.

In the [PDB:1LTH] entry, the estimated electrostatic potential for the allosteric and active site have different values, with 1.22 and -8.09 *kT/e*, respectively, for the R state and similar values for the T state (1.36 and -5.73 *kT/e *respectively). This case matches the concept that active sites may bind more polar or charged molecules, while the allosteric site may bind more drug-like ligands [2]. When averaging these values over the corresponding member pockets, the standard deviation is close in magnitude to the values obtained, being 2.04 *kT/e *for the allosteric site cluster and -3.73 *kT/e *for the active site cluster 2. As discussed above, the electrostatic potential estimations tend to vary largely from structure to structure and thus are hard to extrapolate across the different proteins in a family.

Briefly, in this protein family we found the active site to match expected characteristics of biologically relevant pockets, being very well conserved both in terms of sequence and structure. The allosteric site, despite being very well conserved in terms of structure, does not appear to be conserved at the sequence level.

#### ADP Ribosylation factor 1

**Figure 6:**
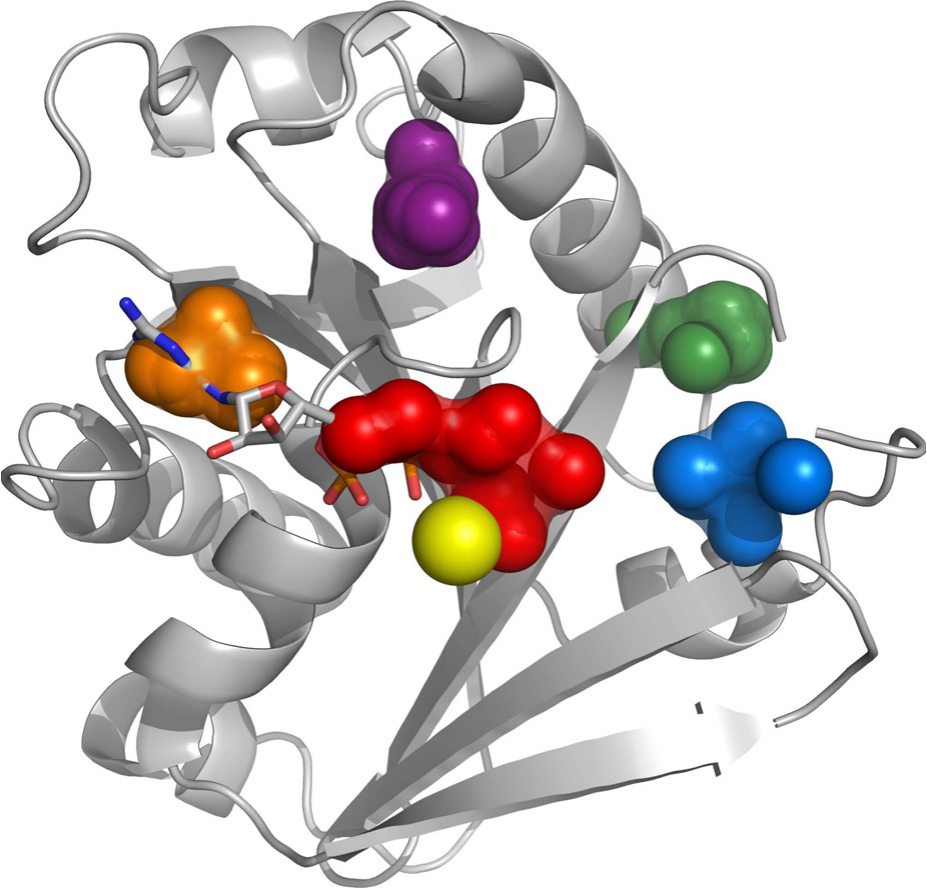
**Arf pocket clusters**. Human ADP-Ribosylation factor 1 structure ([PDB:1HUR], chain A which corresponds to Pfam Arf domain). The first five pocket clusters of this protein family are displayed by showing the centers of mass of the member pockets in 'spheres' representation (cluster 1: red, 2: green, 3: orange, 4: blue, 5: purple). The allosteric activator GDP is displayed in 'sticks' representation next to cluster 1, the Mg ion is colored yellow. Cluster 1 matches the pyrophosphate group of GDP and cluster 3 matches the pyrimidine-imidazole region of the allosteric ligand.

ADP-ribosylation factors (ARFs) are essential and ubiquitous in eukaryotes, being involved in vesicular transport and functioning as activators of phospholipase D and cholera toxin [37]. ARF activity is regulated by the binding and hydrolysis of GTP. The atomic structure [PDB:1HUR] shows the allosteric regulator bound to the protein [37], matching the position of cavity clusters 1 and 3 as displayed in Figure 6. Both clusters matching the allosteric site show high levels of structural and sequence conservation as summarized in Table 2. These clusters also tend to be rigid, with clusters 1 and 3 having 63.6% and 76.9% of their pockets significantly rigid, respectively.

**Table 2:** Arf pocket clusters.

Cluster	structural conservation (%)	sequence conservation (%)	% flexible	% rigid
1	62.5	100.0	0.0	63.6
2	87.5	0.0	14.3	14.3
3	81.3	100.0	0.0	76.9
4	43.8	14.3	42.9	42.9
5	93.8	100.0	0.0	60.0

Properties of the principal pocket clusters of the Arf protein family. The structural conservation corresponds to the percentage of representative structures where a pocket of this cluster is present, the sequence conservation represents the percentage of pockets that are significantly conserved at the sequence level. The last two columns correspond to the percentages of significantly flexible or rigid pockets detected in the corresponding cluster.

Cluster 1 matches the pyrophosphate group of GDP and the Mg ion as displayed in Figure 6. This cluster covers 62.5% of the representative structures of this protein family and we found all of the included pockets to be significantly conserved at the sequence level. The numbers for cluster 3 are similar, as 100% of its pockets are significantly conserved at the sequence level and are detected on 81.3% of the representative structures of this protein family. This cluster matches the pyrimidine-imidazole part of GDP as displayed in Figure 6.

We do not know the biological function, if any, of the pockets represented by the rest of the clusters displayed in Figure 6. Although cluster 2 shows a high level of structural conservation covering 87.5% of the family, the pocket is not significantly conserved at the sequence level. Another interesting cluster is number 5, which appears on 93.8% of the structures and has a sequence conservation of 100%. This cluster is also rigid, with 60% of its pockets being significantly rigid, similarly to the clusters matching the allosteric site. It is interesting that cluster 4 shows almost half of its pockets to be significantly rigid and the other half to be significantly flexible as indicated by the values in Table 2. Initially we thought that this could be related to co-crystallized ligands affecting the flexibility of particular pockets through binding, but none of the analyzed structures presented a ligand in this position. We compared two structures corresponding to this family, namely PDB entry [PDB:1Z6X] (where the corresponding pocket is significantly flexible) and [PDB:1FZQ] (where the pocket was found to be sig-nificantly rigid). The rigid pocket in [PDB:1FZQ] was located next to an *α*-helix, while the same region in [PDB:1Z6X] lacked secondary structure presenting a loop-like conformation. It is remarkable that a pocket may be consistently found in two structures of the same family in a region with diverse secondary structure arrangements and levels of local backbone flexibility. Given that flexibility plays an important role in binding affinity [32], structurally conserved pockets may present distinct binding dynamics that can be exploited in the design of highly specific drugs.

### Prediction of allosteric sites

The idea that yet undiscovered allosteric sites may be found in already solved structures has been mentioned in a review by Hardy and Wells [2], in which they show various examples of previously undescribed allosteric sites found by serendipity. This concept, along with the few cases we examined in detail, prompted us to estimate the number of putative allosteric sites that may be found in the structural data set. We defined a simple estimator that consisted in scanning the data set for pairs of pocket clusters that are conserved at the structural level and are at least 8 Å appart (centroids distance). We performed this analysis on 1,128 protein families for which we had at least 5 representative structures. The results are presented in Table 3. A surprisingly large percentage of protein families (90.6%) presents at least one pair of pocket clusters that are both structurally conserved and at least 8 Å apart. A smaller fraction (54%) of protein families displays also a sequence conservation signal. If we also require one of the pockets to match active site annotations [38], the numbers are smaller but relatively large, *i.e. *for the total of 258 protein families in the database with an active site annotation, 207 (80.2%) present another structurally conserved pocket that is located at a distance of at least 8 Å.

**Table 3:** Pairs of conserved distant pocket clusters, allosteric sites prediction.

Conservation thresholds (%)		# protein families			
**structural**	**sequence**	**total**	sequence conserved*	**active site match**	**both**

50	50	1,022	614	207	165
50	75	1,022	484	207	136
75	50	434	264	93	76
75	75	434	207	93	61

Protein families for which we found a pair of structurally conserved pocket clusters at least 8 Å appart of each other. We used 50% and 75% as the structural conservation thresholds for each pocket cluster in each pair combined as shown in the first column with the sequence conservation threshold. *At least one of the pocket clusters was found to have at least 50% or 75% of its pockets significantly conserved at the sequence level. These results cover 1,128 protein families for which we had at least 5 representative structures.

For example, we found such a pair of pocket clusters on the structures corresponding to the PIG-L Pfam family. One of theses structures ([PDB:1Q7T]) corresponds to MshB from *Mycobacterium tuberculosis *and is considered a potential therapeutic target [39]. This protein lacks active site annotation in Pfam [38] or in the Catalytic Site Atlas [27]. However, the group of Baker *et al. *localized the active site when determining the protein's structure [39]. The active site predicted by Baker and co-workers matches cluster 2 in our predictions and is highly conserved both at the structure (100%) and sequence (80%) levels. Cluster 1 in this family is represented in all structures, although it shows no sign of sequence conservation. In the solved structure, it appears close to the location of a ligand referred to as a crystallization artifact [39]. Moreover, while the active-site-matching cluster shows a strong average electrostatic potential of -7.89 *kT/e*, cluster 1 presents a much more neutral average value of -1.23 *kT/e*. Cluster 3 presents also high levels of structural conservation (80%) and, in addition, of sequence level conservation (75%). Both clusters 1 and 3 would be interesting candidates for virtual screening in the search for an allosteric effector ligand.

On the large-scale perspective, the large amounts of putative allosteric sites we have counted may be an overestimation. Many of these cases may represent pockets that are merely the consequence of structural or functional requirements in other regions of the protein. It would be interesting to test for functional links between these regions [8,11]. However, many protein families do not necessarily perform functions that are associated to a certain pocket, such as the ADP Ribosylation factor discussed above. In these cases, it would not be necessary to find a pair of conserved pockets at a certain distance, since the regulatory site may be a pocket while the protein activity itself may not take place *via *such a structural feature.

## Conclusions

We have developed a simple methodology to estimate the structural conservation of protein pockets, based on their position and size, and have applied it to the large amount of publicly available structural data, covering 4,258 distinct protein families and 22,312 protein structures. The analysis reported here indicates that the presence of structurally conserved pockets is a common feature across protein families and, in some cases, is accompanied by distinctive pocket characteristics in terms of sequence conservation, flexibility or electrostatic potential. Although correlations between the latter properties and structural conservation appear to be low in general, there is, as expected, a higher correlation between pocket structure and sequence conservation for active sites than for other types of annotated or putative ligand-binding pockets. Conserved pockets that lack annotation may represent new opportunities for drug discovery approaches such as virtual screening. In antimicrobial-discovery projects, for example, knowledge of the extent to which a putative ligand-binding site is present across a given protein family (*i.e. *orthologous proteins in a range of species or genuses) can be applied to the design of broad-spectrum drugs, as well as in dealing with drug toxicity, given that an ideal binding site for an antimicrobial would be present in proteins across many pathogenic species but not in a human homolog. In turn, additional pocket properties such as those considered here may be used for fine selection among pockets with the required level of structural conservation. Thus, we have shown specific examples illustrating that sequence conservation and electrostatic potential may be in some instances used to distinguish active sites from allosteric sites, the latter having a lower sequence-conservation signal and a more neutral potential in these examples. The data generated in this study is available upon request.

## Methods

### Structural data set

We organized the large number of structures available at the Protein Data Bank (PDB) [14] in protein families by querying all protein sequences, derived from the atomic coordinates of PDB entries, against the Pfam database (release 23.0) [26]. We performed all sequence-based queries by means of the HMMER software suite [40].

To ensure the quality of the structural data and its relevance to our study, we applied a set of filtering criteria. We evaluated the stereochemistry of protein structures using the PROCHECK program [41] and removed from our data set entries with a G-Factor value lower than -1.00. In the case of structures solved by crystallographic techniques, we also required a resolution of at least 3.0 Å. Entries not solved by Nuclear Magnetic Resonance (NMR) lacking a resolution value were discarded, independently of the technique used. We also discarded Pfam entries of type 'Motif' or 'Repeat', keeping only types 'Family' and 'Domain' that were assigned to structural regions spanning at least 30 residues.

All structures in our data set were parsed and organized according to the Pfam entry they were assigned to. However, given the bias present in the PDB [28], a protein family would be poorly represented by a redundant set containing all related structures. To partially remediate this, we clustered the structures in each Pfam entry according to sequence identity (95%) using complete-linkage hierarchical clustering. For each of the obtained clusters, the structure with the best resolution was chosen as the group representative.

### Predicting ligand-binding pockets at the protein-family level

To compare the spatial positions of potential ligand-binding pockets in different structures of the same protein family, we first superimposed the representative structures to a common reference by means of the MAMMOTH program [29]. The protein with the longest sequence in the family was taken as reference for the structural fit. If length alone failed to select a single reference structure, we used resolution as the second selection parameter.

We proceeded to predict putative ligand-binding pockets on the fitted structures using the LIGSITEcs program [19]. Residues were assigned to pockets according to a common distance criterion, which includes all residues within 8 Å of the pre-calculated pocket's center of mass [19]. Note that the standalone version of the LIGSITEcs program we used is different from the LIGSITEcsc version also mentioned in [19], as the former does not incorporate residue conservation as a parameter.

At this point, for each protein family we had a group of superimposed representative protein structures for which the location of putative ligand-binding pockets had been predicted. We then grouped together pockets found consistently in the same position in different representative structures of the same protein family using the clustering method described below. The calculated clusters were finally ranked according to the average size of their member pockets and the percentage number of family representatives featuring the pocket (coverage).

### Clustering of pockets

We clustered putative ligand-binding pockets found in different representative structures, previously superimposed, of a protein family using a modified version of a previously described clustering algorithm [42]. In this case, the elements to cluster are the centers of mass of pockets and the metric used to define distances between elements is the Euclidean distance. The clustering algorithm makes no distinction between pockets belonging to different, superimposed structures or to the same structure. Given that the degree of structural diversity among representatives of a protein family varies across protein families, the threshold to the metric for the definition of neighbor elements was chosen to be family specific, as described in the Results section. Unlike the previous implementation of the algorithm, cluster selection is not made by straight neighbor counting but by the sum of neighbor pocket sizes, as predicted by LIGSITEcs [19].

The algorithm outline is as follows: (1) the center of mass of each predicted pocket (element) in the set of representative structures of a protein family is assigned a parameter corresponding to the size of the pocket; (2) the Euclidean distance between every pair of elements is calculated; (3) a threshold distance is applied to identify the neighbors of each element in the family; (4) each element is scored by the sum of its size parameter with those of all its neighbors; (5) the element with the highest score is chosen as the center of a cluster, which is formed by all its neighbors; (6) the members of the selected cluster are removed from the pool of elements and the procedure is repeated until the pool is empty; (7) clusters are ranked according to their score, calculated as(1)

where *Score*_*c *_is the cluster's score, *n*_*c *_is the number of pockets in the cluster, *size*_*i *_is the size of member pocket *i *and *Str*_*c *_is the cluster's coverage of the protein family or structural conservation, as described in Results. *Str*_*c *_is computed by(2)

where *m *is the total number of representative structures for the corresponding protein family and *n*_*r *_is the number of representative structures with at least one pocket present in the cluster.

### Sequence conservation

There are multiple methods to estimate sequence conservation starting from a multiple sequence alignment (MSA) [30]. We estimated the degree of positional conservation for every residue in our structure data set by the following procedure: (1) We aligned all sequences in the Pfam 'full' MSA [26] using the HMMALIGN program [40] and the corresponding HMM profile. (2) We computed the entropy of each position of the alignment by means of the AL2CO program [30], activating the program option that weights each sequence to partially compensate MSA composition bias [43]. (3) The entropy values for each position in the MSA were inverted (higher score means higher degree of conservation) and normalized by the observed standard deviation. (4) For each Pfam entry we stored the conservation scores obtained using the HMM profile positions as a reference. (5) We aligned each of our structures to the corresponding HMM profile and assigned the previously computed conservation scores to each residue.

To test if a pocket was significantly conserved at the sequence level, we compared the sequence conservation values obtained for all the residues within 8 Å of the center of mass of the pocket with those for all residues in the structure by applying the Wilcoxon-Mann-Whitney non-parametric test. We defined as significant those cases where the *p*-value <= 0.05.

### Electrostatic potential

We estimated the electrostatic protential at the center of mass of the protein ligand-binding pockets by means of the DELPHI software suite [35], which provides finite-difference solutions to the Poisson-Boltzmann equation. First, we added hydrogen atoms to each structure in our data set using the REDUCE program [44], then proceeded to estimate the electrostatic potential by means of the DELPHI program, with default parameters.

### Protein backbone flexibility

We estimated protein backbone flexibility from normalized B factors as previously described [21,22,45]. For each C*α *atom in the structure, the flexibility is equivalent to its B factor after normalization by equation 3.(3)

where *< B >*is the average over all C*α *atoms in the structure and *σ *(*B*) is the standard deviation. We then define a residue's relative backbone flexibility as the B' value of its C*α*.

For NMR entries, which lack B factors, we calculated the root-mean-square fluctuation (RMSF) of each C*α *atom over the ensemble of NMR models [46]. RMSF values may be in turn converted to pseudo B factors [47], by(4)

We tested for pockets that differed significantly from the complete structures in terms of their flexibility. For each pocket in each structure, we compared the values obtained for residues within 8 Å of the center of mass of the pocket to those for all residues in the structure by applying the Wilcoxon-Mann-Whitney non-parametric test. If the values for the pocket were significantly higher, we marked the pocket as 'flexible' and if they were significantly lower, we marked the pocket as 'rigid'. We defined as significant those cases where the *p*-value *<*= 0.05.

### Mapping active site residues to pockets

Active-site residue predictions by Pfam [38] usually involve between one and three residues. We combined this sequence level information with the structural prediction of pockets on protein structures by mapping active sites to predicted pockets. For each structure, we marked as active site the pocket that included the majority of predicted active site residues.

In many cases, more than a single pocket contained one or more active site residues. We marked these cases as ambiguous, to distinguish them from cases where the mapping was unambiguous, *i.e. *all active site residues contained in a single pocket.

## Authors' contributions

XD and AP conceived the study and wrote the manuscript. AP carried out the computational work. All authors read and approved the final manuscript.

## References

[B1] Pardee AB Regulatory molecular biology Cell Cycle 2006 5 8 846 852 16552190 10.4161/cc.5.8.2634

[B2] Hardy JA Wells JA Searching for new allosteric sites in enzymes Curr Opin Struct Biol 2004 14 6 706 715 10.1016/j.sbi.2004.10.009 15582395

[B3] Cui Q Karplus M Allostery and cooperativity revisited Protein Sci 2008 17 8 1295 1307 10.1110/ps.03259908 18560010 PMC2492820

[B4] Laskowski RA Gerick F Thornton JM The structural basis of allosteric regulation in proteins FEBS Lett 2009 583 11 1692 1698 10.1016/j.febslet.2009.03.019 19303011

[B5] Han Y Malak H Chaudhary AG Chordia MD Kingston DG Bane S Distances between the paclitaxel, colchicine, and exchangeable GTP binding sites on tubulin Biochemistry 1998 37 19 6636 6644 10.1021/bi9719760 9578547

[B6] Mitra A Sept D Taxol allosterically alters the dynamics of the tubulin dimer and increases the flexibility of microtubules Biophys J 2008 95 7 3252 3258 10.1529/biophysj.108.133884 18621813 PMC2547448

[B7] Freire E Can allosteric regulation be predicted from structure? Proc Natl Acad Sci USA 2000 97 22 11680 11682 10.1073/pnas.97.22.11680 11050192 PMC34332

[B8] Lockless SW Ranganathan R Evolutionarily conserved pathways of energetic connectivity in protein families Science 1999 286 5438 295 299 10.1126/science.286.5438.295 10514373

[B9] Balabin IA Yang W Beratan DN Coarse-grained modeling of allosteric regulation in protein receptors Proc Natl Acad Sci USA 2009 106 34 14253 14258 10.1073/pnas.0901811106 19706508 PMC2732817

[B10] Kidd BA Baker D Thomas WE Computation of conformational coupling in allosteric proteins PLoS Comput Biol 2009 5 8 e1000484 10.1371/journal.pcbi.1000484 19714199 PMC2720451

[B11] Daily MD Gray JJ Allosteric communication occurs via networks of tertiary and quaternary motions in proteins PLoS Comput Biol 2009 5 2 e1000293 10.1371/journal.pcbi.1000293 19229311 PMC2634971

[B12] Hardy JA Lam J Nguyen JT O'Brien T Wells JA Discovery of an allosteric site in the caspases Proc Natl Acad Sci USA 2004 101 34 12461 12466 10.1073/pnas.0404781101 15314233 PMC514654

[B13] Morita K Kawana K Sodeyama M Shimomura I Kagechika H Makishima M Selective allosteric ligand activation of the retinoid X receptor heterodimers of NGFI-B and Nurr1 Biochem Pharmacol 2005 71 1-2 98 107 10.1016/j.bcp.2005.10.017 16288995

[B14] Berman HM Westbrook J Feng Z Gilliland G Bhat TN Weissig H Shindyalov IN Bourne PE The Protein Data Bank Nucleic Acids Res 2000 28 235 242 10.1093/nar/28.1.235 10592235 PMC102472

[B15] Stuart AC Ilyin VA Sali A LigBase: a database of families of aligned ligand binding sites in known protein sequences and structures Bioinformatics 2002 18 200 201 10.1093/bioinformatics/18.1.200 11836232

[B16] Marti-Renom MA Rossi A Al-Shahrour F Davis FP Pieper U Dopazo J Sali A The AnnoLite and AnnoLyze programs for comparative annotation of protein structures BMC Bioinformatics 2007 8 Suppl 4 S4 10.1186/1471-2105-8-S4-S4 17570147 PMC1892083

[B17] Brady GP Stouten PF Fast prediction and visualization of protein binding pockets with PASS J Comput Aided Mol Des 2000 14 4 383 401 10.1023/A:1008124202956 10815774

[B18] Glaser F Morris RJ Najmanovich RJ Laskowski RA Thornton JM A method for localizing ligand binding pockets in protein structures Proteins 2006 62 2 479 488 10.1002/prot.20769 16304646

[B19] Huang B Schroeder M LIGSITEcsc: predicting ligand binding sites using the Connolly surface and degree of conservation BMC Struct Biol 2006 6 19 10.1186/1472-6807-6-19 16995956 PMC1601958

[B20] Huang B MetaPocket: a meta approach to improve protein ligand binding site prediction OMICS 2009 13 4 325 330 10.1089/omi.2009.0045 19645590

[B21] Bartlett GJ Porter CT Borkakoti N Thornton JM Analysis of catalytic residues in enzyme active sites J Mol Biol 2002 324 105 121 10.1016/S0022-2836(02)01036-7 12421562

[B22] Yuan Z Zhao J Wang ZX Flexibility analysis of enzyme active sites by crystallographic temperature factors Protein Eng 2003 16 2 109 114 10.1093/proeng/gzg014 12676979

[B23] Nicola G Smith CA Abagyan R New method for the assessment of all drug-like pockets across a structural genome J Comput Biol 2008 15 3 231 240 10.1089/cmb.2007.0178 18333758 PMC2660599

[B24] Bate P Warwicker J Enzyme/non-enzyme discrimination and prediction of enzyme active site location using charge-based methods J Mol Biol 2004 340 2 263 276 10.1016/j.jmb.2004.04.070 15201051

[B25] Chothia C Lesk AM The relation between the divergence of sequence and structure in proteins EMBO J 1986 5 4 823 826 3709526 10.1002/j.1460-2075.1986.tb04288.x PMC1166865

[B26] Finn RD Tate J Mistry J Coggill PC Sammut SJ Hotz HR Ceric G Forslund K Eddy SR Sonnhammer ELL Bateman A The Pfam protein families database Nucleic Acids Res 2008 36 Database D281 D288 18039703 10.1093/nar/gkm960 PMC2238907

[B27] Porter CT Bartlett GJ Thornton JM The Catalytic Site Atlas: a resource of catalytic sites and residues identified in enzymes using structural data Nucleic Acids Res 2004 32 Database D129 D133 10.1093/nar/gkh028 14681376 PMC308762

[B28] Xie L Bourne PE Functional coverage of the human genome by existing structures, structural genomics targets, and homology models PLoS Comput Biol 2005 1 3 e31 10.1371/journal.pcbi.0010031 16118666 PMC1188274

[B29] Ortiz AR Strauss CEM Olmea O MAMMOTH (matching molecular models obtained from theory): an automated method for model comparison Protein Sci 2002 11 11 2606 2621 10.1110/ps.0215902 12381844 PMC2373724

[B30] Pei J Grishin NV AL2CO: calculation of positional conservation in a protein sequence alignment Bioinformatics 2001 17 8 700 712 10.1093/bioinformatics/17.8.700 11524371

[B31] Iwata S Kamata K Yoshida S Minowa T Ohta T T and R states in the crystals of bacterial L-lactate dehydrogenase reveal the mechanism for allosteric control Nat Struct Biol 1994 1 3 176 185 10.1038/nsb0394-176 7656036

[B32] Teague SJ Implications of protein flexibility for drug discovery Nat Rev Drug Discov 2003 2 7 527 541 10.1038/nrd1129 12838268

[B33] Daily MD Gray JJ Local motions in a benchmark of allosteric proteins Proteins 2007 67 2 385 399 10.1002/prot.21300 17295319

[B34] Carlson HA Protein flexibility and drug design: how to hit a moving target Curr Opin Chem Biol 2002 6 4 447 452 10.1016/S1367-5931(02)00341-1 12133719

[B35] Rocchia W Sridharan S Nicholls A Alexov E Chiabrera A Honig B Rapid grid-based construction of the molecular surface and the use of induced surface charge to calculate reaction field energies: applications to the molecular systems and geometric objects J Comput Chem 2002 23 128 137 10.1002/jcc.1161 11913378

[B36] Garvie EI Bacterial lactate dehydrogenases Microbiol Rev 1980 44 106 139 6997721 10.1128/mr.44.1.106-139.1980 PMC373236

[B37] Amor JC Harrison DH Kahn RA Ringe D Structure of the human ADP-ribosylation factor 1 complexed with GDP Nature 1994 372 6507 704 708 10.1038/372704a0 7990966

[B38] Mistry J Bateman A Finn RD Predicting active site residue annotations in the Pfam database BMC Bioinformatics 2007 8 298 10.1186/1471-2105-8-298 17688688 PMC2025603

[B39] McCarthy AA Peterson NA Knijff R Baker EN Crystal structure of MshB from Mycobacterium tuberculosis, a deacetylase involved in mycothiol biosynthesis J Mol Biol 2004 335 4 1131 1141 10.1016/j.jmb.2003.11.034 14698305

[B40] Eddy SR Profile hidden Markov models Bioinformatics 1998 14 9 755 763 10.1093/bioinformatics/14.9.755 9918945

[B41] Laskowski RA Rullmannn JA MacArthur MW Kaptein R Thornton JM AQUA and PROCHECK-NMR: programs for checking the quality of protein structures solved by NMR J Biomol NMR 1996 8 4 477 486 10.1007/BF00228148 9008363

[B42] Daura X Gademann K Jaun B Seebach D van Gunsteren WF Mark AE Peptide Folding: When Simulation Meets Experiment Angewandte Chemie International Edition 1999 38 1-2 236 240 10.1002/(SICI)1521-3773(19990115)38:1/2<236::AID-ANIE236>3.0.CO;2-M

[B43] Henikoff S Henikoff JG Position-based sequence weights J Mol Biol 1994 243 4 574 578 10.1016/0022-2836(94)90032-9 7966282

[B44] Word JM Lovell SC Richardson JS Richardson DC Asparagine and glutamine: using hydrogen atom contacts in the choice of side-chain amide orientation J Mol Biol 1999 285 4 1735 1747 10.1006/jmbi.1998.2401 9917408

[B45] Carugo O Argos P Accessibility to internal cavities and ligand binding sites monitored by protein crystallographic thermal factors Proteins 1998 31 2 201 213 10.1002/(SICI)1097-0134(19980501)31:2<201::AID-PROT9>3.0.CO;2-O 9593193

[B46] Wilmanns M Nilges M Molecular replacement with NMR models using distance-derived pseudo B factors Acta Crystallogr D Biol Crystallogr 1996 52 Pt 5 973 982 10.1107/S0907444996003393 15299607

[B47] Willis BTM Pryor AW Thermal vibrations in crystallography 1975 Cambridge University Press

